# Cognitive and academic outcome following cranial irradiation and chemotherapy in children: a longitudinal study

**DOI:** 10.1054/bjoc.1999.0912

**Published:** 2000-01-18

**Authors:** V A Anderson, T Godber, E Smibert, S Weiskop, H Ekert

**Affiliations:** Dept. Psychology, University of Melbourne, Parkville, Victoria, 3052, Australia; Departments of Psychology, Royal Children's Hospital, Parkville, Victoria, 3052, Australia; Haematology/Oncology, Royal Children's Hospital, Parkville, Victoria, 3052, Australia

**Keywords:** childhood leukaemia, cranial irradiation, intellectual educational sequela

## Abstract

Cranial irradiation therapy (CRT) and chemotherapy are associated with neurobehavioural deficits. Many studies have investigated late effects of these treatments, but few have evaluated changes in abilities over time. This study employed a longitudinal design to map abilities following these treatments. Three groups of children were studied: Group 1 (*n* = 35): children treated with CRT (18 Gy) + chemotherapy, aged 5 years or less at time of diagnosis; Group 2 (*n* = 19): children treated with chemotherapy alone, aged 5 years or less at time of diagnosis; Group 3 (*n* = 35): healthy children. All children were aged 7–13 years at time of initial assessment, with no pre-diagnosis history of neurologic, developmental, or psychiatric disorder. Intellectual and educational abilities were evaluated twice: T1, not less than 2 years post-treatment, and T2, 3 years later. Group 1 achieved poorest results at T1, with comparison groups performing similarly. At T2 group differences were maintained. For verbal skills differences remained stable. Group 1 exhibited deterioration on non-verbal and processing tasks, while comparison groups showed improved abilities. Group 1 exhibited increases in literacy skills, with educational intervention predicting progress. Results suggest cumulative deficits in non-verbal and information processing skills for children treated with CRT + chemotherapy, with other deficits remaining relatively stable over time. Improved literacy skills suggest that gains can occur with remediation. © 2000 Cancer Research Campaign


*British Journal of Cancer* (2000) **82**(2), 255–262
© 2000 Cancer Research Campaign
Article no. bjoc.1999.0912

### Cognitive and academic outcome following cranial irradiation and chemotherapy in children: a longitudinal study

**VA Anderson[1], T Godber[2], E Smibert[3], S Weiskop[1] and H Ekert[3]**

[1]Dept. Psychology, University of Melbourne, Parkville, Victoria 3052, Australia; Departments of [2]Psychology and [3]Haematology/Oncology, Royal Children's Hospital, Parkville, Victoria 3052, Australia

**Summary** Cranial irradiation therapy (CRT) and chemotherapy are associated with neurobehavioural deficits. Many studies have investigated late effects of these treatments, but few have evaluated changes in abilities over time. This study employed a longitudinal design to map abilities following these treatments. Three groups of children were studied: Group 1 ($n$ = 35): children treated with CRT (18 Gy) + chemotherapy, aged 5 years or less at time of diagnosis; Group 2 ($n$ = 19): children treated with chemotherapy alone, aged 5 years or less at time of diagnosis; Group 3 ($n$ = 35): healthy children. All children were aged 7–13 years at time of initial assessment, with no pre-diagnosis history of neurologic, developmental, or psychiatric disorder. Intellectual and educational abilities were evaluated twice: T1, not less than 2 years post-treatment, and T2, 3 years later. Group 1 achieved poorest results at T1, with comparison groups performing similarly. At T2 group differences were maintained. For verbal skills differences remained stable. Group 1 exhibited deterioration on non-verbal and processing tasks, while comparison groups showed improved abilities. Group 1 exhibited increases in literacy skills, with educational intervention predicting progress. Results suggest cumulative deficits in non-verbal and information processing skills for children treated with CRT + chemotherapy, with other deficits remaining relatively stable over time. Improved literacy skills suggest that gains can occur with remediation.
© 2000 Cancer Research Campaign

**Keywords:** childhood leukaemia; cranial irradiation; intellectual educational sequelae

Cranial irradiation therapy (CRT), in combination with a variety of chemotherapeutic regimens, has now been used in the treatment of childhood acute lymphoblastic leukaemia (ALL) for over 25 years. Prior to the introduction of CRT, these children had a life expectancy of less than 1 year, but today approximately 70% of childhood victims survive the disease. While CRT is no longer universally employed in treatment protocols for ALL, there remain large numbers of childhood survivors who are now in continuous remission and quality of life issues are of increasing concern. Research suggests that many children treated with CRT and chemotherapy experience cognitive, educational and behavioural difficulties, with the degree of deficit associated with a range of treatment-based and psychosocial factors (Brouwers et al, 1990; Anderson et al, 1994; Jankovic et al, 1994). The majority of studies link such neurobehavioural deficits specifically to the administration of CRT, or a possible synergistic effect of CRT and chemotherapy in combination (Goff et al, 1980; Ivnik et al, 1981; Gamis et al, 1991; Hallberg et al, 1991; Waber et al, 1995). There is little documentation of the possible sequelae of chemotherapy alone, although some recent research has suggested that similar impairments may occur (Brown et al, 1992; Kaufmann et al, 1996). As many treatment protocols now omit CRT in preference to chemotherapy, clarification of the relative impact of the various treatments is of clinical relevance.

*Received 31 March 1998*
*Revised 27 August 1999*
*Accepted 14 September 1999*

*Correspondence to:* VA Anderson

While it is now well established that neurobehavioral impairments do occur in association with CRT and chemotherapy administered in childhood, there is uncertainty with respect to progression of these problems. It remains unclear whether these deficits stabilize or diminish with time since treatment, or if there may be an ongoing decline in abilities. While histological and radiographic studies have revealed evidence of delayed neuropathology following CRT and chemotherapy (McIntosh et al, 1977; Constine, 1991; Fernandez-Bouzas et al, 1992; Paakko et al, 1992; Valk et al, 1992; Bakke et al, 1993; Matsumoto et al, 1995; Moore, 1995), there has been little systematic examination of related changes in neurobehavioural skills over time. A handful of early studies found no evidence of intellectual and educational decline following therapy (Tamaroff et al, 1982; Moehle et al, 1985; Mulhern et al, 1991). Other longitudinal research indicates that declines in these abilities do occur (Meadows et al, 1981; Stehbens et al, 1983), but may be unique to specific risk factors, such as younger age at treatment (Jannoun and Chessels, 1987) or higher doses of CRT (Silber et al, 1992), or that they may take some time to 'emerge' following treatment (Rubenstein et al, 1990).

While these results do support a decline in abilities with time, interpretation of such findings is problematic. Test–retest practice effects on test measures, which often involve increases of up to ten IQ points per administration, must be considered, and may mask presence of true deterioration of abilities. Such factors are particularly relevant for studies where children are administered the same test on several occasions (e.g. Jannoun and Chessels, 1987; Mulhearn et al, 1991). Additionally, the possible impact of 'psychosocial' variables, such as gender, quality of the child's environment, educational experience, and availability of educational interventions may result in changes unrelated to treatment factors (Taylor and Alden, 1997). Few studies have considered such factors when interpreting changes in ability in this population.

This study aimed to extend previous research, to document the development of children treated with CRT and chemotherapy more than 5 years post-treatment, while minimizing the confounding effects of varying treatment factors. For irradiated children, only those treated with 18 Gy were included, as (i) the neurobehavioural deficits of higher doses of CRT are now well established; and (ii) this lower dose group is more representative of current treatment practices. Further, only children treated at or before age 5 were examined, as this is considered to be at 'high risk' age group with respect to neurobehavioural impairment. It is considered that the developing central nervous system (CNS) may be particularly vulnerable to toxic agents during this time.

Based on reports which describe residual CNS abnormalities, and sometimes ongoing degeneration following CRT in children, it was predicted that children treated with CRT and chemotherapy would exhibit increasing intellectual and educational difficulties over time when compared to healthy control children. In keeping with the results from previous research, we hypothesized that the greatest impairments would continue to be in non-verbal and information processing skills (attention, speed of processing). While less is known about outcome following treatment with standard chemotherapy protocols, the lack of impairment exhibited by this group in our initial research (Anderson et al, 1994) led us to predict that children administered chemotherapy alone would not experience decline in neurobehavioural abilities, but rather, they would demonstrate developmental trajectories similar to those of healthy control children.

### PATIENTS AND METHODS

### Participants

The children described in this study represent a subset of the total group evaluated in our previous studies (Anderson et al, 1994; Smibert et al, 1996). The present sample comprised three groups of children: Group 1 ($n$ = 35): survivors of ALL, treated with cranial irradiation (18 Gy) and chemotherapy; Group 2 chemotherapy only group ($n$ = 19): survivors of other forms of cancer (no CNS involvement) treated with chemotherapy only; and Group 3 ($n$ = 35): healthy control children. For CRT and chemotherapy only groups, only children in remission since initial treatment, who had completed a single course of therapy were included. Initial assessment occurred no less than 2 years after the cessation of treatment, to ensure children were physically recovered, had returned to school and were leading a relatively normal life. For all groups, children with a premorbid history of developmental, neurological or psychiatric disorder were excluded from the sample.

Children considered for inclusion in the CRT group had been treated for ALL at the Royal Children's Hospital, Melbourne, between 1977 and 1987 according to the ANZCCSG Study (V) protocol (Waters, 1992). Cranial irradiation was administered between 2 and 5 years of age, after children had achieved remission following induction chemotherapy. Each child received a course of cranial irradiation (18 Gy) in combination with four doses of intrathecal methotrexate given at weekly intervals. Children also received two doses of intrathecal methotrexate, prior to irradiation, given on day 1 and day 21 of the chemotherapy regimen.

From the original sample ($n$ = 100), 39 children met the criteria for follow-up: (i) dose of CRT administered = 18 Gy; (ii) age at treatment less than 5 years; and (ii) aged 7–13 years at first assessment. Four eligible children were unable to be contacted.

The chemotherapy only group comprised children with an initial diagnosis of ALL, acute myeloid leukaemia (AML) or solid tumour, with no CNS involvement, treated only with chemotherapy. The composition of the group, in terms of aetiology, is relatively heterogeneous, reflecting the lower survival rate for these conditions in comparison to ALL, and the difficulty in enrolling large numbers of children in longitudinal research. From the original sample of children treated with chemotherapy only ($n$ = 50), 31 children met the criteria for follow-up and 19 agreed to participate. Twelve eligible children had either died in the intervening 3-year period or were unable to be contacted.

All 19 children in Group 2 received systemic (intravenous) chemotherapy, with methotrexate at standard dose (Waters, 1992). The group comprised 2 children with a diagnosis of ALL and 2 children had non-Hodgkin's lymphoma. These children received chemotherapy treatment according to the same protocol as children in the CRT group (Waters, 1992). Five children had acute myeloid leukaemia (AML) and were treated according to the then current AML protocol (Tiedemann et al, 1993). Five had Wilms' tumours, and were treated using the appropriate protocol (Hutson et al, 1983). The remaining four children had diagnoses of rhabdomyosarcoma, Ewing's tumour and hepatoblastoma, and each received treatment according to the then current protocol. Within this group ten children received intrathecal methotrexate (ALL, AML, non-Hodgkin's lymphoma), and nine had intravenous chemotherapy alone.

The healthy comparison group was initially recruited from schools within the Melbourne metropolitan area, and the original sample ($n$ = 100) is described in Anderson et al (1994). The healthy comparison group ($n$ = 35) employed in the follow-up study was selected from the original sample, to match the CRT group as closely as possible for age, gender and SES. Only children aged under 17 years at T2 were invited to participate in the follow-up study, due to age requirements for testing.

Demographic and treatment characteristics for the groups included in the follow-up study are provided in Table 1. All children invited to participate in the follow-up study agreed to do so.

Statistical comparisons of demographic, intellectual and educational variables for the initial and follow-up samples confirmed that the samples selected for follow-up did not differ significantly from the original samples on these variables. For the chemotherapy only samples, the follow-up group achieved marginally lower intellectual and educational scores at initial assessment. These group differences only reached statistical significance for Spelling. This trend suggests that the chemotherapy only group described in this study may represent a marginally lower functioning sample, than that described in our original study. The impact of this potential sample bias would be to increase the chances of the chemotherapy only group performing more poorly, and thus similarly to CRT group. In our original study, the chemotherapy only group was indistinguishable from healthy controls on intellectual and educational measures (see Anderson et al, 1994).

© 2000 Cancer Research Campaign

**Table 1** Sample characteristics

| | Group 1 (CRT + chemotherapy) | Group 2 (Chemotherapy only) | Group 3 (Healthy controls) |
|---|---|---|---|
| *n* | 35 | 19 | 35 |
| Number of males | 17 | 11 | 18 |
| Age at testing (years) M (s.d.) | 12.9 (1.9) | 14.2 (2.0) | 13.2 (1.8) |
| Socio-economic status* M (s.d.) | 4.9 (1.0) | 4.1 (2.3) | 4.1 (1.1) |
| Time since testing (years) M (s.d.) | 3.1 (1.6) | 4.7 (0.6) | 3.1 (0.3) |
| Age at diagnosis (years) M (s.d.) | 3.0 (1.0) | 4.4 (2.3) | – |
| Time since treatment (years) M (s.d.) | 9.7 (2.6) | 9.8 (1.7) | – |

* Daniel's Scale of Occupational Prestige.

### METHODS

Families were contacted by letter to participate in the study, and required to provide written, informed consent prior to their inclusion in the study, in keeping with hospital ethics requirements. Three years after the original assessment, families with children meeting the revised selection criteria were contacted with an invitation to undergo reassessment. Children were assessed in a single 2-h session by a child psychologist.

The Wechsler Intelligence Scale for Children – Revised (WISC-R: Wechsler, 1974) was employed as a measure of intellectual performance. Individual subtest scaled scores were calculated as well as Full Scale (FSIQ), Verbal (VIQ) and Performance (PIQ) intellectual quotients. The Wide Range Achievement Test – Revised (WRAT-R: Jastak et al, 1984), which includes Reading, Spelling and Arithmetic subtests, provided a measure of educational abilities. Both tests were administered at initial (T1) and follow-up (T2) evaluations.

At T1 and T2 parents and children also completed questionnaires which provided information regarding medical, family and educational factors, and any changes occurring between evaluations. Parental occupations were recorded, with the occupation of the principal breadwinner used to determine socioeconomic status (SES). The Daniel Scale of Occupational Prestige (Daniels, 1983) was used to quantify these data, using a 7-point rating where higher scores denote lower SES.

At the completion of the initial assessment, all participants were provided with copies of an information booklet derived from earlier research with this population (Godber et al, 1993), which outlined strategies to aid school-based learning. Additional intervention was provided according to the level of impairment exhibited by the child. This intervention was categorized as follows, with higher codes reflecting a greater degree of intervention: (1) no feedback; (2) verbal feedback of assessment results to parents; (3) verbal feedback plus recommendations and written report to family only; (4) verbal feedback plus recommendations and written report to family and telephone advice to school; (5) verbal feedback plus recommendations and written report to family and telephone advice to school together with documentation of educational intervention by school. The nature of feedback was consistent, outlining the child's cognitive and educational strengths and weaknesses, and suggesting strategies for intervention based on a compensatory approach (Hartlage et al, 1983), that is using the child's strengths to overcome weaknesses, with detailed information and strategies available in the booklet provided (Godber et al, 1993).

### Statistical analysis

Group differences for demographic and treatment variables were examined using analysis of variance (ANOVA). For intellectual and educational data, repeated measures ANOVAs (Group × Time × Sex) were conducted across the three groups for T1 and T2 results. Where statistical differences were identified post-hoc analyses were employed to determine group differences. Further, non-parametric analyses ($\chi^2$) were performed on cognitive and educational data to investigate the frequency of significant changes in performances from T1 to T2. A significant increase in performance on these measures was defined as a T2 score more than 5 points higher than that achieved at T1. Similarly, a significant decrease was recorded where T2 score was more than 5 points less than that achieved at T1. Where T1 and T2 scores varied by less than 5 points, results were considered stable or unchanged.

**Table 2** Educational interventions implemented between T1 and T2 for the three groups*

| | Group 1 (CRT + chemotherapy) | Group 2 (Chemotherapy only) | Group 3 (Healthy controls) |
|---|---|---|---|
| *n* | 35 | 19 | 35 |
| No verbal feedback *n* (%) | – | – | 22 (62.9) |
| Verbal feedback only *n* (%) | 13 (37.1) | 13 (68.4) | 9 (25.7) |
| Verbal + written report *n* (%) | 8 (22.9) | 1 (5.3) | – |
| Verbal + written report + school liaison *n* (%) | 6 (17.1) | 1 (5.3) | 3 (8.6) |
| Verbal + written report + school liaison + documented intervention by school *n* (%) | 8 (22.9) | 4 (21.0) | 1 (2.8) |

* All participants received a booklet outlining appropriate educational strategies.

© 2000 Cancer Research Campaign

**Table 3** Results for intellectual and educational measures at T1 and T2.

| | Group 1 (*n* = 35) (CRT + chemotherapy) | | Group 2 (*n* = 19) (Chemotherapy only) | | Group 3 (*n* = 35) (Healthy controls) | |
|---|---|---|---|---|---|---|
| | T1 M (s.d.) | T2 M (s.d.) | T1 M (s.d.) | T2 M (s.d.) | T1 M (s.d.) | T2 M (s.d.) |
| *WISC-R:* | | | | | | |
| FIQ M (SD)[a] | 93.2 (13.1) | 91.0 (12.6) | 101.8 (13.6) | 100.7 (13.8) | 107.3 (12.4) | 107.2 (10.4) |
| VIQ M (SD)[a,b] | 91.9 (15.1) | 89.9 (14.0) | 101.1 (14.9) | 98.5 (13.2) | 105.6 (12.0) | 101.7 (10.9) |
| PIQ M (SD)[a,c] | 96.3 (12.4) | 94.1 (11.4) | 101.8 (12.7) | 103.4 (15.3) | 108.0 (12.8) | 112.5 (10.3) |
| *WRAT-R* | | | | | | |
| Reading M (SD)[a,c] | 86.1 (17.4) | 91.5 (16.9) | 96.1 (19.1) | 93.6 (15.8) | 102.1 (15.2) | 101.9 (11.6) |
| Spelling M (SD)[a] | 85.7 (16.7) | 90.9 (15.4) | 95.2 (18.2) | 95.4 (16.3) | 101.8 (14.0) | 101.5 (14.3) |
| Arithmetic M (SD)[a] | 86.7 (12.5) | 86.9 (15.4) | 100.3 (16.4) | 93.9 (15.0) | 99.5 (11.1) | 97.7 (14.6) |

[a] Significant group difference. [b]Significant time difference. [c] Significant group × time interaction.

A series of hierarchical multiple regressions was conducted to investigate predictors of intellectual and educational outcome. Predictors were entered into analyses as follows: intellectual ability (FSIQ) was entered in the first block, with group membership, T1–T2 interval, SES, gender and level of educational intervention entered in subsequent steps.

### RESULTS

Table 1 provides treatment and demographic characteristics of the three groups. No significant group differences were found for age at testing, time interval between assessments, gender or SES. The group breakdown for educational interventions received from T1 to T2 is provided in Table 2. Examination of these data indicated that, as expected, children experiencing more severe intellectual and educational difficulties were more likely to receive higher levels of intervention, and associated with this trend, the CRT group also received more intervention.

### Between group comparisons

Means and standard deviations (s.d.) for intellectual and educational variables are provided in Table 3. Repeated measures analysis of variance was performed to investigate group and time

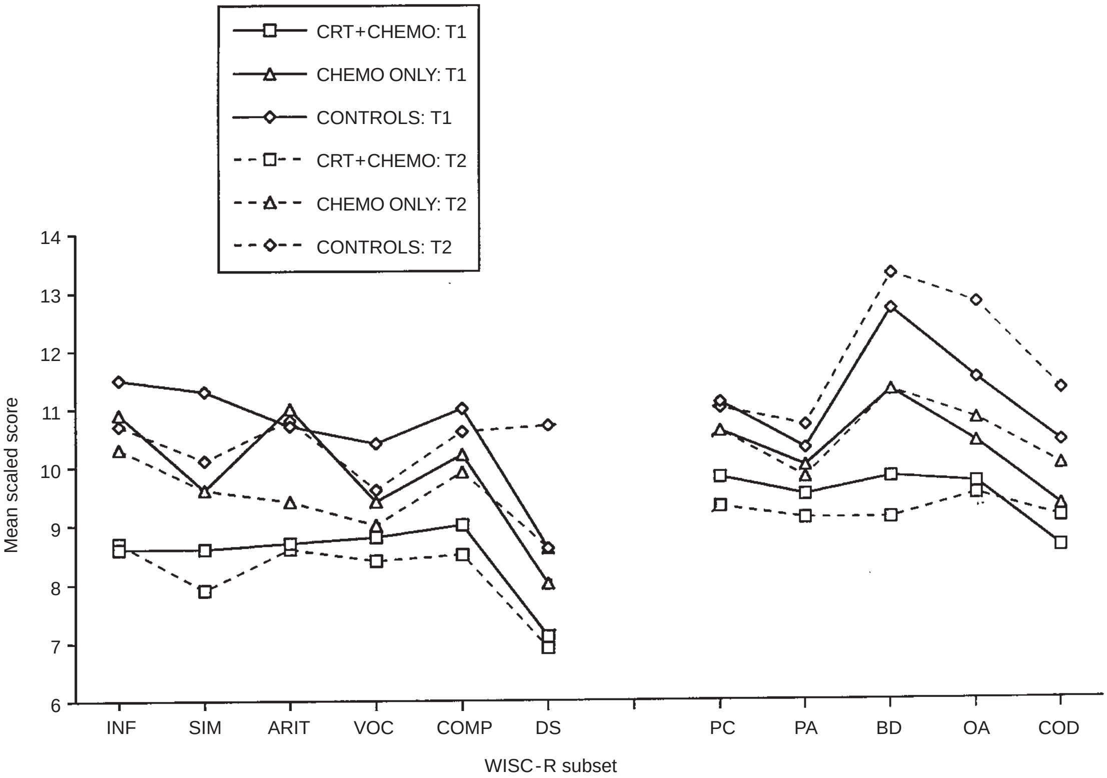

**Figure 1** Comparison of intellectual profiles (WISC-R) for the three groups at T1 and T2. Abbreviations: Information (INF), Similarities (SIM), Arithmetic (ARIT), Vocabulary, (VOC), Comprehension (COMP), Digit Span (DS), Picture Completion (PC), Picture Arrangement (PA), Block Design (BD), Object Assembly (OA), Coding (COD)

© 2000 Cancer Research Campaign effects for summary IQ and educational measures. For intellectual measures, significant Group differences were identified for all summary measures (FSIQ: $F(2,86) = 14.04$, $P < 0.001$: VIQ: $F(2,86) = 9.32$, $P < 0.001$; PIQ: $F(2,86) = 15.0$, $P < 0.001$), with post-hoc analyses indicating that the CRT group performed significantly more poorly than comparison groups. A significant Time effect was detected for VIQ ($F(1,86) = 8.88$, $P < 0.01$), with scores for all groups decreasing from T1 to T2. This finding is unexpected and may be related to the psychometric and cultural parameters of the test, rather than to a true decline in verbal skills. No significant gender effects were identified. A Group × Time interaction ($F(2,87) = 5.42$, $P < 0.01$) was detected for PIQ, with chemotherapy only and controls showing increases in scores over time, possibly due to test practice effects. The CRT group, in contrast, recorded a mean decrease of 2.3 IQ points in PIQ from T1 to T2.

Intellectual profiles for three groups are illustrated in Figure 1, showing mean scaled scores for each subtest at T1 and T2. Repeated measures MANOVAs were conducted for Verbal and Performance scale subtests separately. For Verbal subtests, which tap linguistic abilities and verbal knowledge, significant main effects were detected for both Group and Time (Group: Pillais criterion = 0.43, $P < 0.001$; Time: Pillais criterion = 0.17. $P = 0.01$). Univariate F tests detected significant Group differences on all verbal subtests ($P < 0.001$), with the exception of Vocabulary. Significant Time effects were evident for Similarities ($F(1,86) = 4.04$, $P < 0.05$) and Vocabulary ($F(1,86) = 5.69$, $P < 0.05$), with all groups improving their scores from T1 to T2, and for Digit Span ($F(1,86) = 6.51$, $P < 0.01$) where comparison groups exhibited an increase in scaled scores, but the CRT group recorded a decrease in performance. Only Digit Span, a measure of information processing capacity, showed a significant interaction effect ($F(2,86) = 5.73$, $P < 0.01$), with the CRT group showing a decrease in scaled scores from T1 to T2, while the other two groups improved their performances.

For Performance subtests, tapping visual skills, processing speed, planning and problem solving, significant effects were found for Group (Pillais criterion = 0.32, $P = 0.01$), with no main effect of Time and no interaction effect. Univariate F test results showed Group differences ($P < 0.01$) for all Performance scale subtests except Picture Arrangement, with the CRT group consistently achieving lowest scores. All groups improved over time for the Coding subtest ($F(1,86) = 6.15$, $P < 0.01$), and interactions were found for Block Design ($F(2,86) = 3.28$, $P < 0.05$) and Object Assembly ($F(2,86) = 3.52$, $P < 0.05$), with the CRT group showing a decrease in scores from T1 to T2, compared to a small increase for comparison groups.

On the WRAT-R, repeated measures ANOVA identified a significant Group effect for all subtests (Reading: $F(2,86) = 6.67$, $P < 0.01$; Spelling: $F(2,86) = 7.78$, $P < 0.001$; Arithmetic: $F(2,86) = 8.85$, $P < 0.001$), with the CRT group performing consistently more poorly at both T1 and T2. No significant Time effect was identified for any of the educational measures. A gender effect was found for Spelling only ($F(1,86) = 9.40$, $P < 0.001$), with girls exhibiting better spelling skills overall. A Group × Time interaction was detected for Reading ($F(2,85) = 4.13$, $P < 0.05$), with the CRT group showing some gains from T1 to T2 on this measure, compared to small decreases in scores for comparison groups. This pattern of performance was also noted for Spelling, although the effect was not statistically significant.

Finally, for each child, 'change scores' were calculated for VIQ, PIQ, FSIQ and WRAT-R subtests by comparing T1 and T2 scores, and are illustrated in Table 4. All children were categorized as demonstrating decreased, increased or stable scores over time, as defined above. Chi square results, comparing change scores across groups, showed no significant group differences for FSIQ or VIQ. However, for PIQ, group differences were significant ($\chi^2_4 = 10.18$, $P < 0.05$), with children treated with CRT most likely to record significant declines in performance over time, and, conversely, least likely to demonstrate improved scores. For educational measures, no significant group differences were evident for arithmetic skills or spelling. However, for reading, children with CRT were more highly represented in the 'improved results' category ($\chi^2_4 = 10.59$, $P < 0.05$), with 60% of these children exhibiting a significant increase in reading scores.

### Within group analyses

A series of hierarchical multiple regressions was conducted to investigate predictors of intellectual and educational outcome at T2. Predictors were entered into the analysis as follows: FSIQ: T1, to account for factors effecting children's performance prior to initial testing, Group, T1–T2 interval, SES, gender, and level of educational intervention. The results of these analyses are summarized in Table 5. For intellectual variables, the regression equations employed were able to explain approximately two-thirds of the variance, with overall intellectual abilities at T1 highly predictive

**Table 4** Percentage of children demonstrating significant changes[a] in performance from T1 at T2

| | Group 1 ($n$ = 35) (CRT + chemotherapy) | | Group 2 ($n$ = 19) (Chemotherapy only) | | Group 3 ($n$ = 35) (Healthy controls) | |
|---|---|---|---|---|---|---|
| | Decrease $n$ (%) | Increase $n$ (%) | Decrease $n$ (%) | Increase $n$ (%) | Decrease $n$ (%) | Increase $n$ (%) |
| *WISC-R:* | | | | | | |
| FIQ | 13 (37.1) | 6 (17.1) | 7 (36.8) | 4 (21.1) | 8 (22.9) | 8 (22.9) |
| VIQ | 13 (37.1) | 7 (20.0) | 9 (47.4) | 7 (36.8) | 19 (54.3) | 6 (7.1) |
| PIQ* | 14 (40.0) | 6 (17.1) | 5 (26.4) | 7 (36.8) | 4 (11.4) | 16 (45.6) |
| *WRAT-R* | | | | | | |
| Reading* | 7 (20.0) | 21 (60.0) | 7 (36.8) | 2 (10.5) | 12 (34.3) | 14 (40.0) |
| Spelling | 6 (17.1) | 20 (57.1) | 5 (26.3) | 8 (42.2) | 10 (28.6) | 9 (25.7) |
| Arithmetic | 2 (5.7) | 12 (34.3) | 10 (52.6) | 2 (10.5) | 19 (54.3) | 9 (25.7) |

*$P < 0.05$. [a]Significant change is defined as follows: decrease, T2 score is more than 5 points below T1 score; increase, T2 score is more than 5 points above T1 score.

**Table 5** Predictors of intellectual and educational outcome at T2 following cranial irradiation and chemotherapy

| Predictor variables | | FSIQ | VIQ | PIQ | WRAT-R | WRAT-S | WRAT-A |
|---|---|---|---|---|---|---|---|
| FSIQ (T1) | Beta | 0.78 | 0.71 | 0.74 | 0.44 | 0.53 | 0.70 |
| | *t* value | 12.18 | 9.45 | 8.64 | 3.53 | 3.76 | 6.07 |
| | *P* | 0.001 | 0.001 | 0.001 | 0.001 | 0.001 | 0.001 |
| Group | Beta | 2.85 | 11.15 | 4.51 | –1.02 | –0.18 | 1.70 |
| | *t* value | 2.30 | 0.81 | 2.80 | –0.44 | –0.08 | 0.78 |
| | *P* | 0.02 | NS | 0.006 | NS | NS | NS |
| Time since treatment | Beta | –1.74 | –0.88 | –2.60 | –0.65 | –0.20 | –1.11 |
| | *t* value | –2.06 | –0.89 | –2.32 | 0.40 | –0.12 | –0.73 |
| | *P* | 0.04 | NS | 0.02 | NS | NS | NS |
| SES | Beta | –0.50 | –1.66 | 1.22 | –1.10 | –1.15 | 1.05 |
| | *t* value | –0.70 | –2.00 | 1.29 | –0.80 | –0.85 | 0.82 |
| | *P* | NS | 0.05 | NS | NS | NS | NS |
| Gender | Beta | –0.18 | –0.63 | –0.06 | 4.20 | 6.93 | 2.50 |
| | *t* value | –0.13 | –0.37 | –0.03 | 1.50 | 2.50 | 1.00 |
| | *P* | NS | NS | NS | NS | 0.01 | NS |
| Intervention | Beta | –0.50 | –0.16 | –0.87 | –2.44 | –3.26 | –0.80 |
| | *t* value | –0.80 | –0.22 | –1.05 | –2.03 | –2.74 | –0.71 |
| | *P* | NS | NS | NS | 0.05 | 0.008 | NS |
| | $R^2$ | 0.79 | 0.70 | 0.65 | 0.36 | 0.39 | 0.46 |

of results at T2. For FSIQ and PIQ, group membership (CRT group) and longer time since treatment were predictive of poorer outcome, reflecting slower speed of processing and non-verbal skills. For VIQ higher SES was related to higher T2 scores. Level of educational intervention and gender were unrelated to intellectual performance 5 years post-treatment.

Regression analyses for the WRAT-R subtests accounted for less of the overall variance (36–46%), suggesting that future research may need to consider a broader range of predictor variables. Once again, intellectual ability was a strong predictor of educational ability at T2. For Reading and Spelling ability, level of educational intervention was a significant predictor of performance at T2, with greater educational intervention related to improvements in performance. Gender also predicted Spelling ability, with females scoring more highly on these tasks. Group membership, SES, and time since treatment were not predictive of outcome for any of the educational measures. For arithmetic ability none of the predictor variables included in the regression model had an impact on outcome.

### DISCUSSION

The present study aimed to investigate change in intellectual and educational skills over time for survivors of childhood cancers treated prior to age 5 years. Three groups were compared: those treated with CRT and chemotherapy, those treated with chemotherapy alone, and healthy controls. Groups were similar with respect to age at testing, gender and socioeconomic status. At initial evaluation, not less than 2 years post-treatment, significant differences were identified across groups. The 'CRT group' performed most poorly on all measures, and those treated with 'chemotherapy only' achieved results similar to healthy controls, consistent with previous research. The results of the CRT group on intellectual measures fell two-thirds of a standard deviation below the test mean on average. While this does not represent a severe intellectual impairment, such a deficit is of clinical significance, and would be expected to reduce the capacity of these children to function adequately within their environment.

To establish the predicted deterioration over time following CRT and chemotherapy treatments, it was necessary to identify a Group × Time interaction for outcome measures. Thus, not only should treated children perform poorly at initial evaluation, but their development trajectories from T1 to T2 should be flatter than those observed for healthy controls. Such a pattern of results would support the presence of an increasing gap between the normal development exhibited by comparison groups, and that of the CRT group. At follow-up evaluation this pattern of interactions was present for some variables, but it was not consistently identified, failing to support an interpretation of generally slowed development or deterioration in skills. Similarly, there was no evidence of 'recovery' or catchup of abilities over time for the CRT group.

All groups recorded a small decline in VIQ scores, which tap linguistic competence and verbal intelligence. Analysis of subtest performances, as illustrated in Figure 1, indicates that this decline was particularly marked for tests tapping expressive language skills (Similarities, Vocabulary), where all groups recorded poorer scores at T2. With the exception of the Digit Span subtest, this pattern of lower scores was reflected in all verbal subtests, and may represent psychometric limitations of the test employed, or perhaps cultural factors. These results do not indicate a differential fall off in verbal abilities associated with the administration of CRT. In contrast, for the Digit Span subtest, a measure of auditory processing capacity, a Group × Time interaction effect was identified. Analysis of results showed that chemotherapy only and control groups showed improved age-scaled scores from T1 to T2. In contrast, the CRT group exhibited a significant decline in these scores. These data suggest that, in addition to suffering an initial impairment in information processing skills, children treated with CRT and chemotherapy may exhibit a slowed rate of development of these skills.

Similar trends were identified for PIQ, with the CRT group recording slightly lower scores overall at follow-up, and the chemotherapy only and healthy controls exhibiting a corresponding increase in their scores. The increased Performance IQ scores of the latter groups may be expected due to the known practice effects on the IQ measure (Wechsler, 1974). Further examination of trends for

 © 2000 Cancer Research Campaign individual subjects for PIQ suggested that there were significantly more children in the CRT group who showed a decline in test scores (T2 5 points less than T1 (CRT: 40.0%; chemotherapy only: 26.4%; controls: 11.4%)), and conversely, less exhibited a score increase (T2 5 points more than T1 (CRT: 17.1%; chemotherapy only: 36.8%; controls: 45.6%). This decrease in scores exhibited by the CRT group may not necessarily reflect a true deterioration, but rather is likely to represent slower than expected development in non-verbal abilities and information processing skills tapped by the Performance Scale. Interestingly, this pattern of greater 'decline' in scores at an individual level was not so marked for other measures.

These findings are not consistent with a generalized decline in abilities or a global lag in development associated with CRT and chemotherapy. Rather, specific areas of ability were observed to be more susceptible to time effects. Non-verbal skills and information processing, which have been previously identified as areas of greatest difficulty following treatment in young children (Copeland et al, 1985, 1988; Rourke, 1987; Cousens et al, 1988; Rogers et al, 1992; Smibert et al, 1996; Anderson et al, 1997), show poorest development over time, suggesting a cumulative pattern of cognitive impairment. In contrast, verbal abilities maintained development for both treatment groups.

Contrary to expectations of ongoing deterioration, the CRT group exhibited greater than expected improvements in reading and spelling, in contrast to comparison groups which recorded age appropriate increments. These improvements may be associated with both the level of ability of the child and the educational interventions implemented following T1 assessment. The test employed in this study (WRAT-R Reading) measures single word reading only, and may not necessarily generalize to other aspects of reading such as comprehension and fluency, and thus uncertainty remains with respect to the full functional implications of these results. However, to our knowledge no other longitudinal study examining residual deficits following CRT and chemotherapy has attempted to document these factors, or even report whether such interventions have occurred. Our results do indicate a positive response to the provision of feedback regarding the child's intellectual and educational strengths and weaknesses and details regarding appropriate intervention strategies. Greater improvement occurred where written information was available to both parents and schools. The nature of the deficits detected in the initial study suggest that these children treated with CRT and chemotherapy are able to learn, but may do so more slowly than other children. The implication from these findings is that appropriate intervention may minimize educational deficits and reduce the development of secondary psychosocial problems. An alternative explanation is that these educational gains may reflect a 'delay' or developmental lag associated with treatment, where children show initial difficulties, but 'catch up' to their peers over time. Such a position is not supported by intellectual outcomes indicating stable development at best.

These findings are consistent with developmental models purporting the susceptibility of the immature brain. While neuroanatomical studies provide evidence for ongoing CNS changes following early brain damage, our results argue for a similar association for cognitive development. The observation of cumulative deficits in specific cognitive domains (that is, information processing, non-verbal abilities) suggests that skills which are in a critical phase of development during treatment may be particularly susceptible to disruption (Dennis, 1989). Results also support previous research identifying greatest impairments when CRT is included in treatment protocols. In this study children treated with standard chemotherapy protocols were indistinguishable from healthy control children at both T1 and T2, suggesting no detectable detrimental effects associated with their treatment. When CRT is added to the treatment protocol, intellectual and educational deficits occur, with results suggesting an impairment of clinical significance (two-thirds of a standard deviation below expected mean). The conclusion may be drawn that either CRT alone, or a synergistic effect of CRT and chemotherapy, is associated with neurobehavioural sequelae in young children.

In conclusion, our findings indicate that children treated with CRT (18 Gy) and chemotherapy prior to age 5 years are at risk for ongoing intellectual and educational difficulties post-treatment. While some of these deficits remain constant over time (language skills, verbal knowledge), others increase (information processing, non-verbal abilities), reflecting a failure to develop as expected even many years post-treatment. This pattern was not exhibited for children treated with chemotherapy alone, who were largely indistinguishable from healthy controls. Further, provision of information regarding children's cognitive and educational strengths and weaknesses was noted to be associated with improvement in literacy skills, suggesting that appropriate intervention may ameliorate the negative effects of treatment. Future studies are needed to further investigate the efficacy of intervention following CRT and chemotherapy in children.

### ACKNOWLEDGEMENTS

This research was supported by the Royal Children's Hospital Research Foundation and the Anti Cancer Council of Victoria, Australia.

### REFERENCES

Anderson VA, Smibert E, Ekert H and Godber T (1994) Intellectual, educational and behavioral sequelae following cranial irradiation and chemotherapy. *Arch Dis Child* **70**: 476–483

Anderson VA and Moore C (1995) Age at injury as a predictor of outcome following pediatric head injury. *Child Neuropsych* **1**: 187–202

Anderson VA, Godber T, Smibert E and Ekert H (1997*a*) Neurobehavioural sequelae following cranial irradiation in children: an analysis of risk factors. *Pediatr Rehab* **1**: 63–76

Anderson VA, Morse SM, Klug G, Catroppa C, Haritou F, Rosenfeld J and Pentland L (1997*b*) Predicting recovery from head injury in school-aged children: a prospective analysis. *J Int Neuropsychol Soc* **3**: 568–580

Bakke S, Fossen A, Storm-Mathiesen I and Lie S (1993) Long-term cerebral effects of CNS chemotherapy in children with acute lymphoblastic leukemia. *Pediatr Hem Oncol* **10**: 267–270

Brouwers P and Poplack D (1990) Memory and learning sequelae in long-term survivors of acute lymphoblastic leukemia: association with attention deficits. *Am J Pediatr Hem Oncol* **12**: 174–181

Brouwers P, Riccardi R, Fedio R and Poplack D (1985) Long-term neuropsychological sequelae of childhood leukemia: correlation with CT brain scan abnormalities. *J Pediatr* **106**: 723–728

Brown RT, Madan-Swain A, Lambert RG, Lambert RG, Sexon S and Ragab A (1992) Chemotherapy for acute lymphoblastic leukemia: cognitive and academic sequelae. *J Pediatr* **121**: 885–889

Constine L (1991) Late effects of radiation therapy. *Pediatrician* **18**: 37–48

Copeland DR, Fletcher JM, Pfefferbaum-Levine B, Jaffe N, Reid H and Maor M (1985) Neurological sequelae of childhood cancer in long term survivors. *Pediatrics* **75**: 745–753

Copeland DR, Dowell RE, Fletcher JM, Bordeaux JD, Sullivan MP, Jaffe N et al (1988) Neuropsychological effects of childhood cancer treatment. *J Child Neurol* **3**: 53–62

© 2000 Cancer Research Campaign 

Cousens P, Ungerer JA, Crawford JA and Stevens M (1988) Cognitive effects of childhood leukemia therapy: a case for four specific deficits. *J Pediat Psychol* **16**: 475–488
Daniel A (1983) *Power, Privilege and Prestige: Occupations in Australia.* Longman-Cheshire: Melbourne
Dennis M (1989) Language and the young damaged brain. In: *Clinical Neuropsychology and Brain Function: Research, Measurement and Practice*, Boll T and Bryant B (eds), pp. 89–123. Am Psychol Assoc: Washington
Fernandez-Bouzas A, Jimenez HR, Zamudio JV, Alonso-Vanegas M and Guerra RM (1992) Brain calcifications and dementia in children treated with radiotherapy and intrathecal methotrexate. *J Neurosurg Sci* **36**: 211–214
Gamis AS and Nesbit ME (1991) Neuropsychologic (cognitive) disabilities in long-term survivors of childhood cancer. *Pediatrician* **18**: 11–19
Godber T, Anderson V, Smibert E and Ekert H (1993) *Overcoming Learning Difficulties: A Handbook for the Parents and Teachers of Children who Have been Treated for Leukemia.* Anti-Cancer Council of Victoria: Melbourne
Goff JR, Anderson HR and Cooper PF (1980) Distractibility and memory deficits in long-term survivors of acute lymphoblastic leukemia. *Dev Beh Pediatr* **1**: 153–163
Hartlage LC and Terzlow CF (1983) The europsychological basis of educational intervention. *J Learn Dis* **16**: 521–528
Hallberg FE, Kramer JH, Moore IM, Wara W, Mattha K and Ablin A (1992) Prophylactic cranial irradiation dose effects on late cognitive function in children treated for acute lymphoblastic leukemia. *Int J Radiation Onco Biol Phys* **22**: 13–16
Hudspeth W and Pribram K (1990) Stages of brain and cognitive maturation. *J Educ Psychol* **82**: 881–884
Hutson JM, Kent M, Ekert H and Waters KD (1983) The treatment of Wilms' tumour: results from Royal Children's Hospital, Melbourne, 1967–1977. *J Ped Surgery* **18(3)**: 235–239
Ivnik RJ, Colligan RC, Obertz SW and Smithson W (1981) Neuropsychological performance among children in remission from acute lymphocytic leukemia. *Dev Beh Pediatr* **2**: 29–34
Jankovic M, Brouwers P, Vaisecchi MG, Van Veldhuizen A, Huisman J, Kamohius R, Kingma A et al (1994) Association of 1800 cGy cranial irradiation with intellectual function in children with acute lymphoblastic leukemia. *Lancet* **344**: 224–227
Jannoun L and Chessels JM (1987) Long-term psychological effects of childhood leukemia and its treatment. *Pediatr Hem Oncol* **4**: 293–308
Jastak JS and Jastak S (1984) *The Wide Range Achievement Test – Revised*. Jastak: Wilmington, DE
Kaufmann PM, Moore IM, Espy KA and Hutter JJ (1996) Late effects of triple intrathecal chemotherapy on neuropsychological outcome at 24 months post leukemia diagnosis. *J Int Neuropsychol Soc* **2**: 42
Kingma A, Mooyart EL, Kamps WA, Nieuwenhuizen P and Wilmink JT (1993) Magnetic resonance imaging of the brain and neuropsychological evaluation in children treated for acute lymphoblastic leukemia at a young age. *Am J Pediatr Hem Oncol* **15**: 231–238
McIntosh S, Fischer D, Rothman S et al (1977) Intracranial calcifications in childhood leukemia. *J Pediatr* **91**: 909–913
Matsumoto K, Takahashi S, Sato A et al (1995) Leukoencephalopathy in childhood hematopoietic neoplasm caused by moderate-dose methotrexate and prophylactic cranial radiotherapy – an MR analysis. *Int J Radiation Oncol Biol Phys* **32**: 913–918
Meadows AT, Massari DJ, Ferguson J et al (1981) Declines in IQ scores and cognitive dysfunctions in children with acute lymphocytic leukemia treated with cranial irradiation. *Lancet* **ii**: 1015–1018
Moehle KA and Berg RA (1985) Academic achievement and intelligence test performance in children with cancer at diagnosis and one year later. *Dev Beh Pediatr* **6**: 62–64
Moore IM (1995) Central nervous system toxicity of cancer therapy in childhood. *J Pediatr Oncol Nurs* **12**: 203–210
Mulhern RK, Fairclough D and Ochs J (1991) A prospective comparison of neuropsychologic performance of children surviving leukemia who received 18-Gy, or 24-Gy, or no cranial irradiation. *J Clin Oncol* **9**: 1348–1356
Ochs JJ, Parvey LS, Whitaker JN et al (1983) Serial cranial computed tomography scans in children with leukemia given two different forms of central nervous system therapy. *J Clin Oncol* **1**: 793–798
Paakko E, Vainionpaa L, Lanning M et al (1992) White matter changes in children treated for acute lymphoblastic leukemia. *Cancer* **70**: 2728–2733
Rogers J, Britton PG, Kernahan J et al (1992) Cognitive function after two doses of cranial irradiation for acute lymphoblastic leukemia. *Arch Dis Child* **66**: 1245–1246
Rourke BP (1987) Syndrome of non-verbal learning disabilities: the final common pathway of white matter disease/dysfunction. *Clin Neuropsych* **1**: 209–234
Rubenstein CL, Varni JW and Katz ER (1990) Cognitive functioning in long-term survivors of childhood leukemia: a prospective analysis. *Dev Beh Pediatr* **11**: 301–305
Silber JH, Radcliffe J, Peckham V et al (1992) Whole brain irradiation and decline of intelligence: the influence of dose and age on IQ score. *J Clin Oncol* **10**: 1390–1396
Smibert E, Anderson V, Godber T et al (1996) Risk factors for intellectual and educational sequelae of cranial irradiation in childhood acute lymphoblastic leukemia. *Brit J Cancer* **73**: 825–833
Stehbens JA, Kisker CT and Wilson BK (1983) School behavior and attendance during the first year of treatment for childhood cancer. *Psychol Schools* **20**: 23–28
Tamaroff M, Miller DR and Murphy ML (1982) Immediate and long-term post-therapy neuropsychologic performance in children with acute lymphoblastic leukemia treated without central nervous system radiation. *J Pediatr* **101**: 524–529
Tiedemann K, Waters KD, Tauro GP, Tucker D and Ekert H (1993) Results for intensive therapy in childhood acute myeloid leukaemia, incorporating high dose melphalan and autologous bone marrow transplantation in first complete remission. *Blood* **82**: 3730–3738
Valk J and Van Der Knaap MS (1992) Toxic encephalopathy. *Am J Neuroradiation* **13**: 747–760
Waber D, Tarbell N, Fairclough D et al (1995) Cognitive sequelae of treatment in childhood acute lymphoblastic leukemia: cranial radiation requires an accomplice. *J Clin Oncol* **13**: 2490–2496
Waters KD (1992) A randomized clinical trial of modified BFM therapy versus modified high dose asparaginase therapy in childhood acute lymphoblastic leukemia. *Med. Paediatr Oncol* **20**: (abstr 83)
Weschler D (1974) *Wechsler Intelligence Scale for Children – Revised.* Psychol Corp: San Antonio, TX

© 2000 Cancer Research Campaign
